# Vesicular GABA transport expression in kisspeptin cells may contribute to the preovulatory gonadotropin surge

**DOI:** 10.3389/fendo.2026.1807596

**Published:** 2026-04-22

**Authors:** Henrique Rodrigues Vieira, Estela Guidini Ferrufino, Ligia M. M. de Sousa, Guilherme A Alves, Marina G. Martins, Jose Donato, Renata Frazao

**Affiliations:** 1 Departamento de Anatomia, Instituto de Ciências Biomédicas, Universidade de Sao Paulo, Sao Paulo, Brazil; 2 Departamento de Fisiologia e Biofísica, Instituto de Ciências Biomédicas, Universidade de Sao Paulo, Sao Paulo, Brazil

**Keywords:** cre-loxP system, hypothalamic-pituitary-gonadal axis, luteinizing hormone, puberty, *Slc32a1*

## Abstract

**Background:**

The neurotransmitter gamma-aminobutyric acid (GABA) plays a vital role in the modulation of reproductive function by controlling gonadotropin-releasing hormone (GnRH) secretion. Most kisspeptin neurons, which are key regulators of GnRH neurons, coexpress GABA and other neuropeptides. However, whether the expression of the vesicular GABA transporter (Vgat) in kisspeptin cells contributes to the reproductive phenotype of mice remains unclear.

**Methods:**

Brain slices from female vesicular GABA transporter-Cre/GFP (Vgat-Cre/GFP) mice were used to assess GABAergic coexpression in kisspeptin neurons during diestrus and proestrus. Mice carrying conditional ablation of *Slc32a1* (referred to as *Vgat*) in *Kiss1*-expressing cells (Kiss1-Cre/Vgat*^flox/flox^* mice) were generated to study possible effects on reproduction and metabolism.

**Results:**

50-60% of kisspeptin neurons in the anteroventral periventricular and rostral periventricular nuclei are GABAergic. However, the percentage of GABAergic kisspeptin neurons did not differ between proestrus and diestrus stages. Deletion of *Vgat* in kisspeptin cells was sufficient to downregulate *Gnrh1* and *Kiss1r* expression in the rostral periventricular region of female mice. In contrast, *Cartpt* mRNA expression in the mediobasal hypothalamus was downregulated only in male mice compared with controls. Body composition, puberty onset, and fertility were preserved in both sexes; however, female Kiss1-Cre/Vgat*^flox/flox^* mice exhibited significantly lower serum luteinizing hormone levels at proestrus.

**Discussion:**

These findings indicate that *Vgat* expression in kisspeptin neurons may contribute to LH surge generation but is not essential for the onset of puberty or for the maintenance of fertility in either female or male mice.

## Introduction

The gamma-aminobutyric acid (GABA) is classically known as an inhibitory neurotransmitter in the vertebrate brain. However, both inhibitory and excitatory effects of GABA have been demonstrated on the electrical activity of central components of the hypothalamic-pituitary-gonadal (HPG) axis (1, 2). The physiological actions of GABA depend on its ionotropic (GABA_A_ and GABA_C_) and metabotropic (GABA_B_) receptors (3, 4). GABA can either suppress or stimulate gonadotropin-releasing hormone (GnRH) and luteinizing hormone (LH) release, depending on the experimental condition (5–10). While the inhibition of GABA signaling on the pituitary medial stalk is sufficient to induce an increase in GnRH release in prepubertal monkeys, GABA infusion into the medial stalk suppresses GnRH release after puberty onset but not before puberty in female monkeys (7, 8). In adult female rats, treatment with GABA-acetylenic (which inhibits GABA-transaminase activity, increasing GABA concentration) 1 day before ovulation prevents the expected LH surge (5). In ovariectomized females or male rats, intracerebroventricular injection of GABA (4 mM) induces the release of LH (9, 10). Collectively, these studies highlight the dual, highly context-dependent effects of GABA on HPG axis regulation, which vary with age, sex, and the hormonal milieu. Nonetheless, most of this evidence is based on pharmacological approaches that broadly affect GABA signaling across unspecific brain regions, thereby limiting the ability to assign these effects to defined neuronal populations.

GnRH neurons are considered the central modulators of the HPG axis, and their activity depends on multiple inputs besides GABA, such as glutamate and kisspeptins, among other neurotransmitters (2, 5, 11). The fundamental importance of kisspeptins on the modulation of GnRH neuron activity has been extensively reviewed (12–16). To date, it is known that kisspeptin neurons located at the anteroventral periventricular and rostral periventricular nuclei (AVPV/PeN^Kisspeptin^) are considered the essential mediators of the estrogen-mediated positive-feedback mechanism to GnRH cells. In contrast, kisspeptin cells located at the arcuate nucleus of the hypothalamus (ARH^Kisspeptin^) are the hypothalamic GnRH pulse generator necessary for fertility (11, 17–19). In addition, ARH^Kisspeptin^ neurons serve as metabolic sensors to control the HPG axis (20–24). Both GnRH and kisspeptin neurons coexpress GABA receptors (25–33). Importantly, about 75% of AVPV/PeN^Kisspeptin^ neurons and 50% of ARH^Kisspeptin^ neurons express the glutamic acid decarboxylase 67 (GAD67), a GABA-producing enzyme (34). Estimates indicate that 3% to 50% of ARH^Kisspeptin^ neurons also coexpress *Vgat*, depending on the methodological approach used (34, 35). Collectively, these findings support that a substantial proportion of hypothalamic kisspeptin neurons exhibit a GABAergic phenotype.

In mice under normal developmental conditions, GABAergic synaptic transmission to GnRH and kisspeptin neurons decreases during the pubertal transition (36, 37). Interestingly, metabolic distress, such as acute fasting, decreases the frequency of GABAergic inputs to GnRH and ARH^Kisspeptin^ neurons in adult mice (22, 38). Exposure to BPA, an endocrine disruptor, is also sufficient to modulate GABAergic transmission and GnRH release in a time and dose-dependent manner (39–41). In addition, it is noteworthy that estrogen receptor alpha is required for reproduction in Vgat-Cre-expressing cells (42). However, whether GABAergic transmission from kisspeptin cells is necessary for the maturation of the HPG axis and the establishment of fertility remains unknown.

Considering that 50-60% of the AVPV/PeN^Kisspeptin^ neurons are GABAergic and that changes in the GABAergic transmission contribute to the LH surge (6, 34, 43–47), we hypothesized that the gene coding the vesicular GABA transporter (*Slc32a1* here referred to as *Vgat*) expression in kisspeptin cells is essential for the onset of puberty and fertility in both sexes, as well as for generating the preovulatory surge in female mice.

## Materials and methods

### Animals

Vgat-Cre mice (Stock No:028862, Jackson Laboratories, Bar Harbor, ME, USA) were crossed with a Cre-dependent green fluorescent protein (GFP) reporter mouse (Stock No: 026175, Jackson Laboratories, Bar Harbor, ME, USA) to allow the visualization of GABAergic cells (Vgat-Cre/GFP), as previously reported (48). Kiss1-Cre mice (Stock No: 023426, Jackson Laboratories, Bar Harbor, ME, USA) were crossed with mice possessing *loxP* sites flanking exon 2 of the *Slc32a1* (Stock No: 012897, Jackson Laboratories, Bar Harbor, ME, USA). Heterozygous offspring were then crossed to generate mice that were homozygous for *Slc32a1* alleles and that either expressed Cre in a *Kiss1*-dependent fashion (Kiss1-Cre/Vgat*^flox/flox^*) or did not express Cre (control).

At three weeks of age, mice were weaned and genotyped via PCR. The DNA was extracted from tail tips using the REDExtract-N-Amp Tissue PCR Kit (Sigma, St. Louis, MO, USA). Cages with four to five animals were kept in environmentally controlled rooms with a 12 h on/12 h off light cycle (lights on at 06:00 am) and a temperature of 21-23 °C. The Institute of Biomedical Sciences Animals Ethics Committee at the University of São Paulo approved all animal procedures.

### Quantification of kisspeptin-coexpressing Vgat cells

Adult Vgat-Cre/GFP female mice (4–5 months of age) were selected for the experiments. The estrous cycle was assessed daily by evaluating the vaginal lavage. We considered a complete estrous cycle to have occurred when the transition between all estrous phases was observed, starting on the first day of diestrus. Vgat-Cre/GFP mice at the diestrus and proestrus phases of the estrous cycle were anesthetized (5% isoflurane) and euthanized (5:00-6:00 pm). Mice were perfused transcardially with saline, followed by 10% buffered formalin, pH 7.4. After the transcardiac perfusion, we performed the brain and the uterus dissection (n = 5/6 per group) as described (49, 50). The uterus wet weights were recorded. Coronal brain slices (30 μm) were done using a freezing microtome. Four series were collected and stored at −20 °C in a cryoprotectant until processing for immunohistochemistry (49).

To determine the percentage of kisspeptin immunoreactive cells that coexpress *Vgat* at the AVPV/PeN, brain slices were obtained from female Vgat-Cre/GFP mice. Brain slices were rinsed in potassium phosphate buffer saline (KPBS) pH 7.4 and blocked in 3% normal donkey serum (Jackson Laboratories, West Grove, PA) for one hour, followed by incubation with a primary antibody cocktail containing anti-kisspeptin antibody (#AB9754; Millipore; 1:2000) and anti-GFP (# GFP-1020, Aves Labs Inc; 1:5000) in KPBS containing 0.25% Triton X-100 for 24 hours at 4 °C. Sections were subsequently rinsed in KPBS and incubated for 90 min with Alexa Fluor 594- and Alexa fluor 488-conjugated IgG secondary antibodies (1:500, Jackson Laboratories), as described (49). Sections were mounted onto gelatin-coated slides and covered with Fluoromount G (Electron Microscopic Sciences, Hatfield, PA). The photomicrographs were acquired with a Zeiss Axiocam HRc camera coupled to a Zeiss Axioimager A1 microscope (Zeiss, Munich, Germany). Images were digitized using the Axio Vision software (Zeiss). We used the ImageJ Cell Counter software (http://rsb.info.nih.gov/ij/) to manually count neurons at two rostrocaudal levels of the AVPV/PeN (relative to bregma: 0.26 and 0.02 mm). Quantification was performed bilaterally at a representative rostrocaudal level for each region, and values were averaged, as AVPV/PeN are bilateral structures. Bregma was determined according to the Allen Brain Atlas (http://mouse.brain-map.org/).

### RNAscope

To assess the co-expression of *Kiss1* and *Slc32a1* (*Vgat*) transcripts in the AVPV/PeN, RNA *in situ* hybridization was performed. Female mice were transcardially perfused (n=3 per group), and brains were collected as previously described. Coronal sections were processed using the RNAscope^®^ Multiplex Fluorescent V2 kit (ACDBio, #323110) following the manufacturer’s guidelines. Sections were first washed in PBS, incubated at 60 °C for 30 min, and dehydrated through graded ethanol. Tissue pretreatment included incubation in hydrogen peroxide for 10 min at room temperature, followed by Protease III digestion for 30 min at 40 °C. Hybridization was carried out for 2 h at 40 °C using probes targeting *Kiss1* (Mm-Kiss1, #500141, ACDBio) and *Vgat* (Mm-Slc32a1-C3, #319191-C3, ACDBio). Signal detection was achieved using TSA amplification: *Vgat* was revealed with TSA Plus^®^ Fluorescein (1:1,500; Akoya Biosciences, #NEL741001KT), whereas *Kiss1* was detected with TSA Plus^®^ Cy3 (1:1,500; Akoya Biosciences, #NEL744001KT). Sections were counterstained with DAPI, coverslipped using ProLong Gold^®^ antifade reagent (ThermoFisher Scientific, #P36930), and kept at 4 °C protected from light until image acquisition. The photomicrographs were obtained as previously described.

### Assessment of *Vgat*-induced ablation of kisspeptin cells

#### Relative gene expression

Adult Kiss1-Cre/Vgat*^flox/flox^* and control mice were used to determine whether the selective ablation of *Vgat* from kisspeptin cells led to changes in relative gene expression in the rostral periventricular region (RPV), containing the AVPV/PeN, or the mediobasal hypothalamus (MBH), containing the ARH. Mice were anesthetized (5% isoflurane) and euthanized by decapitation (2:00-5:00 pm). We collected hypothalamus from female (diestrus phase of the estrous cycle) and male mice for relative gene expression analyses (2–3 months of age; female, n = 6/9 per group; male, n =6/8 per group). Brain slices and micro-punches were performed based on anatomical references according to the atlas (Allen Brain Institute), as previously described (51). A single midline micro punch of the RPV (bregma 0.38 to 0.02 mm) was obtained from a coronal brain section of 250 µm using an 18-gauge needle. One additional coronal brain slice of 700 µm was cut (relative to bregma -1.22 to -1.94 mm), and bilateral punches of the MBH were collected using an 18-gauge needle.

Following the manufacturer’s instructions, the total RNA from the micro punches was extracted using the PicoPure RNA Isolation Kit (Thermo Fisher Scientific, Cat# KIT0204, Waltham, MA, USA; RID: SCR_008817). An Epoch Microplate Spectrophotometer (Gen5, BioTek, Winooski, VT, USA; RRID: SCR_017317) was used to determine RNA quantity. Reverse transcription was performed by combining 0.25 μg RPV and 0.5 μg MBH of total hypothalamic RNA, the SuperScript II Reverse Transcriptase (Thermo Fisher Scientific), and random primers p(dN)6 (Roche Applied Science, Penzberg, Upper Bavaria, Germany). Real-time polymerase chain reaction (RT-PCR) was performed using a 7500 Real-Time PCR System (Thermo Fisher Scientific) and the Power SYBR Green PCR Master Mix (Thermo Fisher Scientific). All samples were run in duplicate, and negative controls were included on each qPCR plate. Relative mRNA expression was quantified by calculating 2^−ΔΔCt^. Data were normalized to the geometric average of the housekeeping genes *Actb* and *Ppia* and reported as fold change relative to the control group (set to 1.0). The genes and primer set for the RT-PCR analysis are described in **Table 1**. Of note, we observed no significant differences in the average cycle threshold (Ct) values for *Actb* or *Ppia* in the RPV or the MBH, comparing data from Kiss1-Cre/Vgat*^flox/flox^* and control mice (data not shown).

**Table 1:** Primers sequences.

Target name	Forward (5’-3’)	Reverse (5’-3’)
*Actb*	gctccggcatgtgcaaag	catcacaccctggtgccta
*Agrp*	ctttggcggaggtgctagat	aggactcgtgcagccttacac
*Cartpt*	cagtcacacagcttcccgat	cagatcgaagcgttgcaaga
*Esr1*	tggagattcaagtccccaaa	gcagatagggagctggttca
*Gnrh1*	gggttctgccatttgatccac	ccctttgactttcacatcc
*Kiss1*	gattccttttcccaggcatt	ggcaaaagtgaagcctggat
*Kiss1r*	cttcaccgcactcctctacc	acacagtcacataccagcgg
*Npy*	cagatactactccgctctgcg	gggctggatctcttgccata
*Pdyn*	tgtgcagtgaggattcaggatg	accgtcagggtgagaaaagatg
*Pomc*	tagatgtgtggagctggtgc	ccagcgagaggtcgagtttg
*Ppia*	cttcttgctggtcttgccattcc	tatctgcactgccaagactgagt
Slc32a1 *(Vgat)*	gggtcacgacaaacccaaga	gcacgaacatgccctgaatg

#### Body composition

The body weight of Kiss1-Cre/Vgat*^flox/flox^* and control mice was recorded weekly from weaning (postnatal 21 days) to 63 days of age (females n = 7/11 per group; males n = 7/17 per group). Total body fat and lean mass were measured in adult mice (5 months of age, females n = 4/5 per group; males n = 10/14 per group) by time-domain nuclear magnetic resonance using an LF50 body composition analyzer (Bruker, Germany).

#### Reproductive phenotyping

Puberty onset of Kiss1-Cre/Vgat*^flox/flox^* and control mice was assessed by recording the age of vaginal opening and first estrus (n = 8/13 per group) for female mice. In males, the age of balanopreputial separation and testicular descent (n = 7/17 per group) was used as a marker of sexual maturation (49, 52). Body weight was recorded at the time of pubertal events. A subgroup of female Kiss1-Cre/Vgat*^flox/flo^*^x^ and control mice underwent estrous cycle evaluation at 2–3 months of age (female, n = 8/10 per group). Following euthanasia under anesthesia (5% isoflurane) for hypothalamic collection, the gonads were harvested (females, 7/8 per group; males, 6/11 per group), and their wet weights were recorded.

Female or male mice fertility was evaluated by pairing adult Kiss1-Cre/Vgat*^flox/flox^* and control female (n = 8/10 per group) or male mice (n = 6/8 per group) with sexually experienced control animals (1 or 2 females per male) for up to 60 days (49). We recorded the latency to the first litter (days until birth) and the number of pups per litter.

#### Serum hormone assay

Kiss1-Cre/Vgat*^flox/flo^*^x^ female mice were daily adapted to tail-tip blood sampling for 30 days to minimize stress on the experimental day. Blood collection was performed in the afternoon of diestrus or in the afternoon of the proestrus stage (5:00-6:00 pm; n = 6/12 per group). We used a surgical blade to remove a small portion of the tail tip (1 mm), allowing the collection of 5 μl of blood with a pipette. Serum levels of LH were determined using an enzyme-linked immunosorbent assay (ELISA). Briefly, this ELISA used, as a capture antibody, a monoclonal mouse anti-bovine LH beta subunit antiserum (1:2,500; Pablo Ross, UC Davis, 518B7; RRID: AB_2756886), a polyclonal rabbit anti-rat LH detection antibody (AFP240580Rb, NIDDK-NHPP; RRID: AB_2665533), and mouse recombinant LH (AFP-5306A, NIDDK-NHPP) to prepare the standard curve, as described earlier (53, 54). The intra-assay and inter-assay coefficients of variation of the LH ELISA were 4.43%, 7.51%, and 18.65%, respectively.

### Statistical analysis

We used the GraphPad Prism software (GraphPad Prism, San Diego, CA, USA; RRID: SCR_002798) for statistical analysis. The data are presented as the mean ± standard error of the mean (SEM). We used the independent t-test to compare two groups (quantification of kisspeptin-ir cells, gene expression, gonad masses, fat and lean masses, and number of pups per litter). A two-way ANOVA (genotype, age, and their interaction) and Sydak’s *post hoc* test were used to analyze body weight gain data. Non-parametric data (i.e., percentage of kisspeptin-ir cell bodies that co-expressed *Vgat*, age of puberty events, estrous cycle length, latency to the first litter, and LH serum levels) were evaluated using the Mann-Whitney test. Fisher’s exact test was used to determine the fertility rate and the number of mice exhibiting an LH surge during the afternoon of proestrus. Results with *P* values < 0.05 were considered statistically significant.

## Results

### The proportion of kisspeptin-GABAergic neurons is stable during the diestrus and proestrus phases

**Figure 1:**
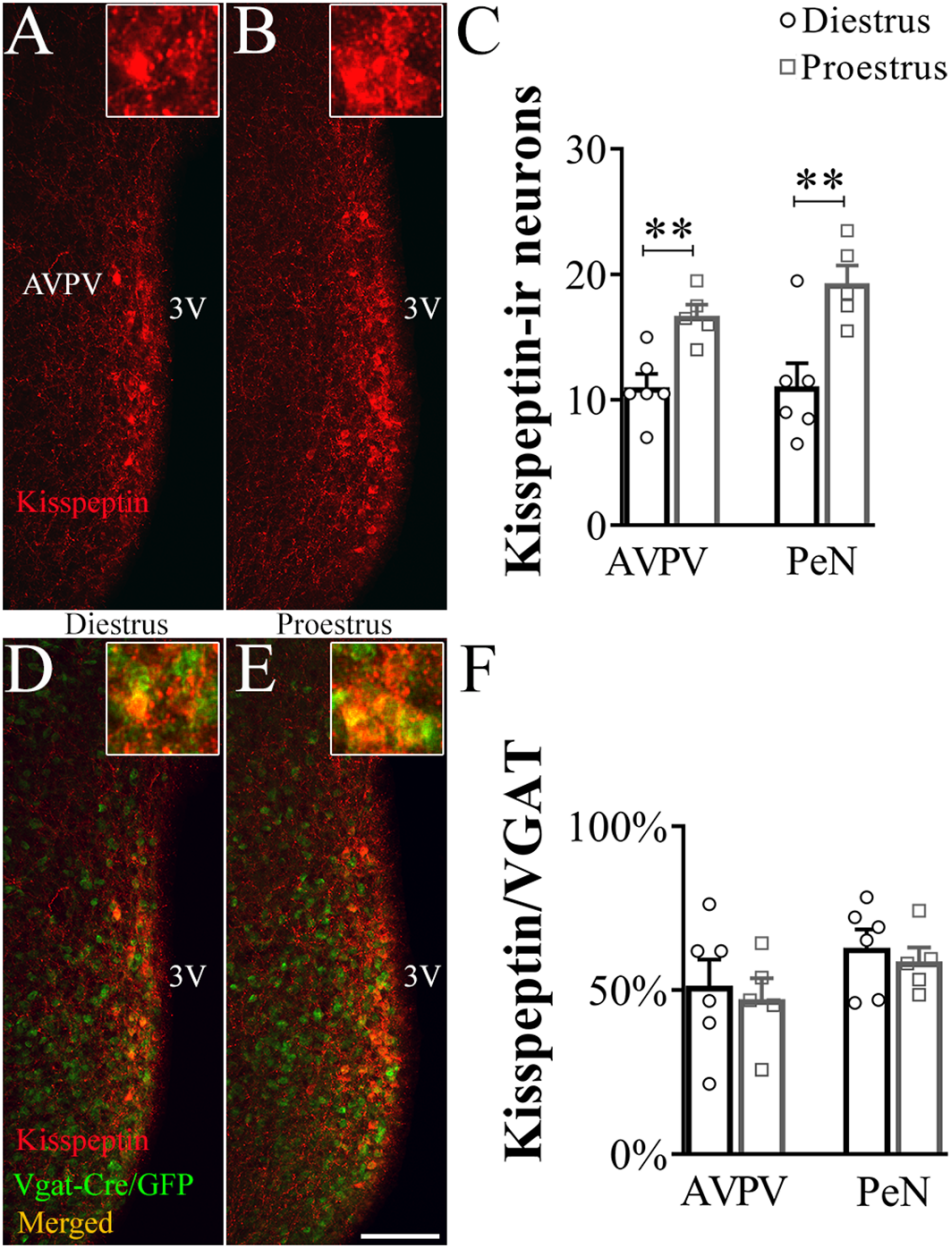
Most AVPV/PeN^Kisspeptin^ neurons are GABAergic. **(A, B)** Epifluorescence photomicrographs of kisspeptin-immunoreactivity (-ir; red) in the anteroventral periventricular nucleus (AVPV) of adult mice on the first day of diestrus **(A)** or at the proestrus day **(B)**. **(D, E)** Epifluorescence photomicrographs showing kisspeptin-ir and GFP-ir (indicating vesicular GABA transporter, *Vgat*) in the AVPV. Vgat-Cre-expressing neurons were identified by GFP (green). Double-labeled cells appear yellowish/orange (merged, insets). **(C, F)** Bar graphs comparing the number of kisspeptin-ir cells **(C)**, or the percentage of kisspeptin-ir cells that coexpress the Vgat-Cre **(F)** in the AVPV and in the rostral periventricular nuclei (PeN; n = 5/6 per group). Abbreviation: 3V, third ventricle. Scale: 50 μm. Values are expressed as mean ± SEM. **P≤0.008.

We selected Vgat-Cre/GFP female mice at the proestrus and diestrus phases for these experiments. The percentage of kisspeptin-ir cell bodies that co-expressed *Vgat* in the AVPV at the diestrus, 51.3 ± 7.9% (5.7 ± 1.0 out of 11.0 ± 1.1 cells), and proestrus, 47.2 ± 6.3% (7.8 ± 1.0 out of 16.7 ± 0.9 cells, *P* = 0.9), or at the PeN at the diestrus, 63.0 ± 5.5% (6.7 ± 0.8 out of 11.1 ± 1.8 cells), and proestrus, 58.7 ± 4.3% (11.2 ± 0.7 out of 19.3 ± 1.4 cells, *P* = 0.8) was similar, independently of the estrous cycle phase (**Figures 1D–F**). As expected, Vgat-Cre/GFP female mice at the proestrus exhibited increased uterine mass (0.16 ± 0.02g, n=5) compared to animals at the diestrus (0.09 ± 0.01g, n=6; *P=*0.02). In addition, the number of kisspeptin-ir cell bodies increased in the AVPV (16.7 ± 0.9 cells) and the PeN (19.3 ± 1.4 cells) at proestrus compared to the diestrus (AVPV, 11.0 ± 1.1 cells, *P=*0.003; PeN, 11.1 ± 1.8 cells; *P=*0.008; **Figures 1A–C**).

### *Vgat* ablation in kisspeptin cells reduces *Gnrh1* and *Kiss1r* mRNA expression

**Figure 2:**
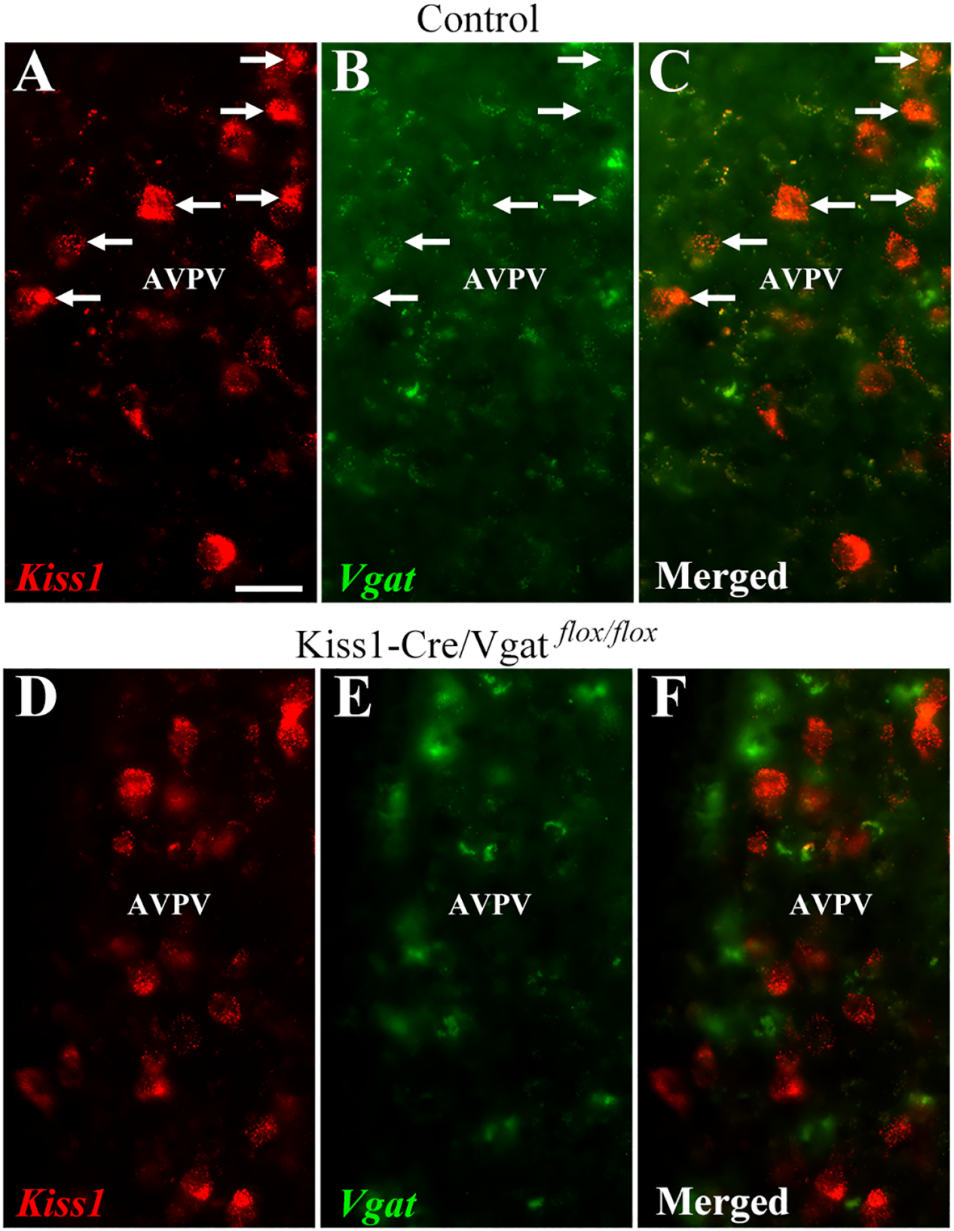
Coexpression between *Kiss1* and *Vgat* mRNA in the hypothalamus of control and Kiss1-Cre/Vgat^flox/flox^ mice. **(A-C)** Epifluorescence photomicrographs showing the colocalization between *Kiss1* mRNA (red) and *Vgat* mRNA (green) in the hypothalamus of control mice. Arrows indicate double-labeling cells. **(D-F)** Epifluorescence photomicrographs showing the lack of colocalization between *Kiss1* mRNA (red) and *Vgat* mRNA (green) in the hypothalamus of Kiss1-Cre/Vgat^flox/flox^ mice (n = 3 per group). Scale bar = 20 µm.

Next, we generated a mouse model in which we selectively ablated *Vgat* in kisspeptin cells (Kiss1-Cre/Vgat*^flox/flox^*). We confirmed that 58% of AVPV/PeN^Kisspeptin^ neurons expressed *Vgat* mRNA in control mice (5.3 ± 2.2 out of 9.3 ± 4.4 cells; **Figures 2A–C**). In contrast, only 9% of AVPV/PeN^Kisspeptin^ neurons in Kiss1-Cre/Vgat*^flox/flox^* mice showed detectable *Vgat* mRNA expression (1.5 ± 0.3 out of 16.8 ± 0.9 cells; **Figures 2D, E**), indicating a significant reduction in the proportion of kisspeptin neurons expressing *Vgat* (*P =*0.002). The number of *kiss1*-expressing cells was similar between control and Kiss1-Cre/Vgat*^flox/flox^* mice (*P* = 0.1).

**Figure 3:**
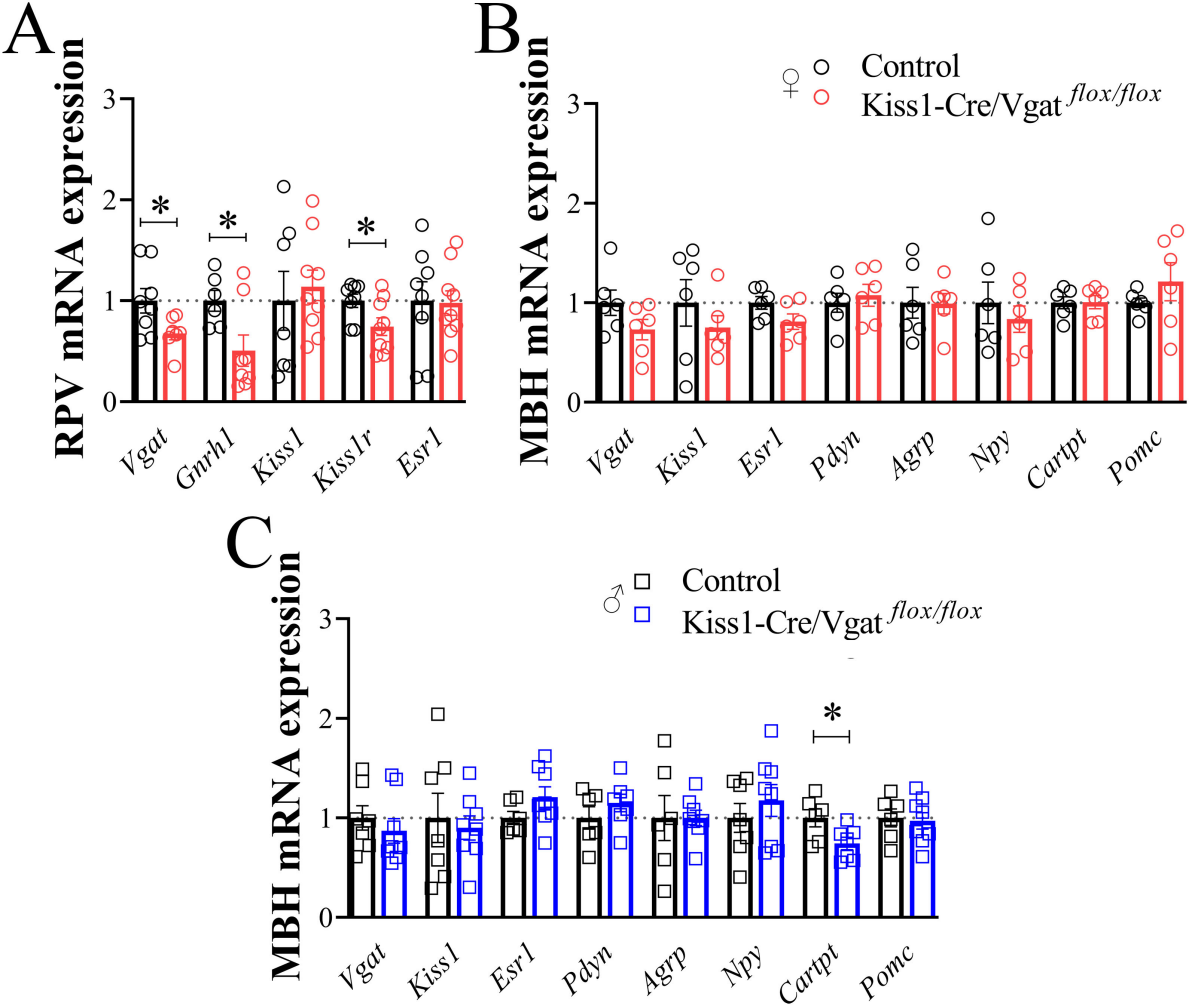
*Gnrh1* and *Kiss1r* mRNA expression are downregulated following *Vgat* ablation from kisspeptin cells. **(A)** Bar graphs comparing *Vgat*, *Gnrh1*, *Kiss1*, *Kiss1r*, and *Esr1* mRNA expression in the punches samples obtained from the rostral periventricular region (RPV) of control and Kiss1-Cre/Vgat*^flox/flox^* female mice (n = 6/9 per group). **(B, C)** Bar graphs comparing *Vgat*, *Kiss1*, *Esr1*, *Pdyn*, *Agrp*, *Npy*, *Cartpt*, and *Pomc* mRNA expression in the punches obtained from the medio basal hypothalamus (MBH) of control and Kiss1-Cre/Vgat*^flox/flox^* female and male mice (n = 6/8 per group). Values are expressed as mean ± SEM. *P<0.05.

To further confirm the *Vgat* deletion, we measured *Vgat* mRNA expression in the RPV and MBH of Kiss1-Cre/Vgat*^flox/flox^* and control mice. *Vgat* mRNA levels were significantly reduced in the RPV of Kiss1-Cre/Vgat^flox/flox^ female mice compared with control mice (**Figure 3A**). In contrast, no significant difference was observed in the MBH (**Figures 3B, C**). We further evaluated whether *Vgat* ablation in kisspeptin cells modulates the expression of genes regulating the reproductive axis and energy balance in female and male mice. Interestingly, *Vgat* ablation in kisspeptin cells was associated with reduced *Gnrh1* and *Kiss1r* expression in the RPV (*P<*0.05; **Figure 3A**). No changes in *Kiss1* and *Esr1* mRNA levels were observed in the RPV (*P>*0.05; **Figure 3A**). In the MBH, we observed no differences in *Kiss1*, *Esr1*, *Pdyn*, *Agrp*, *Npy*, and *Pomc* mRNA expression between Kiss1-Cre/Vgat*^flox/flox^* and control mice (*P>*0.05; **Figures 3B, C**). In male Kiss1-Cre/Vgat*^flox/flox^* mice, *Cartp* mRNA levels in the MBH were significantly reduced compared with control animals (*P<*0.05), whereas *Cartp* levels did not vary in the MBH of Kiss1-Cre/Vgat*^flox/flox^* female mice (**Figures 3B, C**). These results suggest that the conditional *Vgat* ablation in kisspeptin cells is sufficient to modulate *Gnrh1* and *Kiss1r* expression mRNA in the RPV.

### Kiss1-Cre/Vgat^flox/flox^ mice show a slight increase in body weight during development

**Figure 4:**
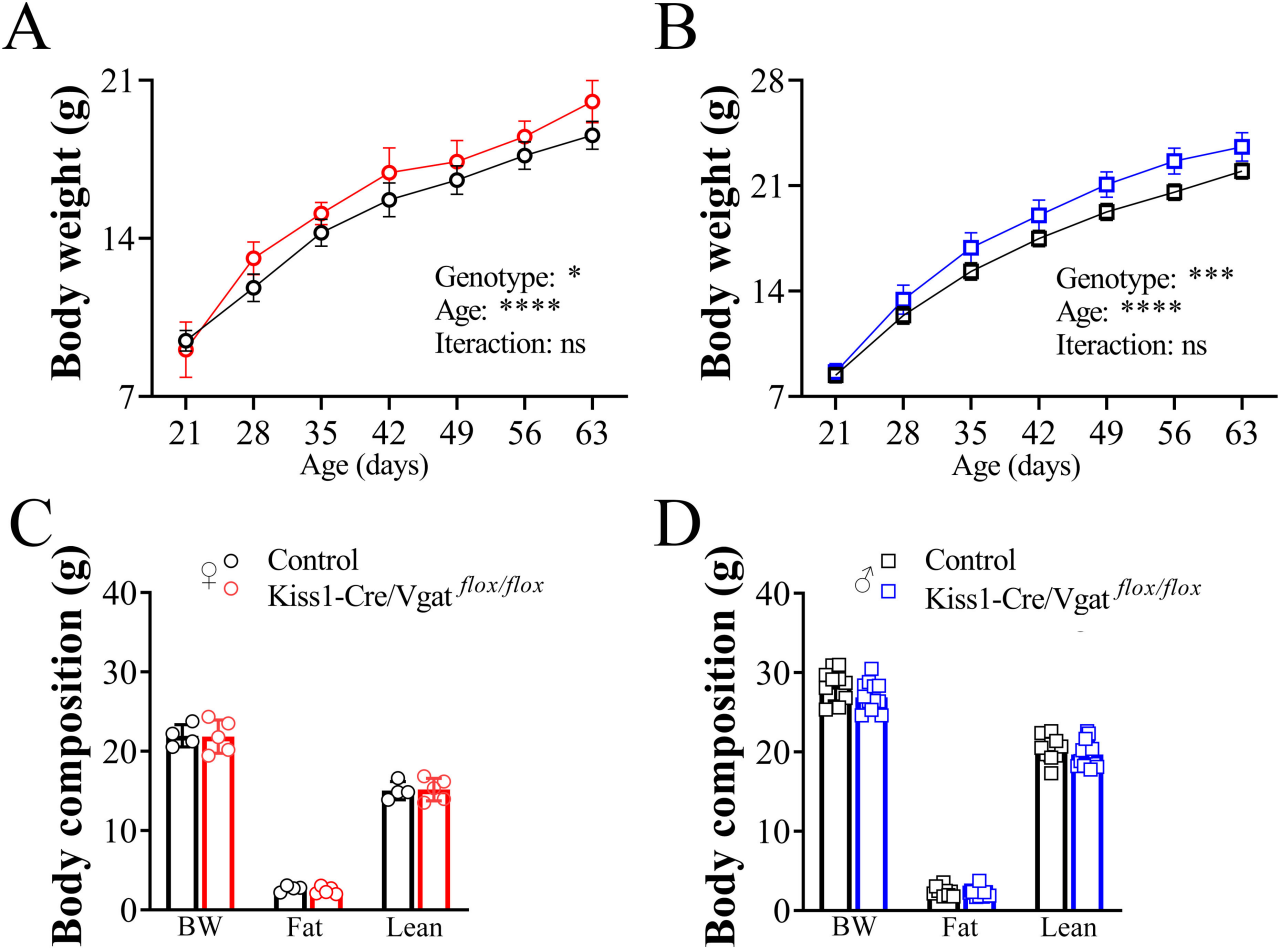
Ablation of vesicular GABA transporter in kisspeptin cells leads to a slight increase in body weight gain throughout development. **(A, B)** Body weight gain in female (A, n = 7/11 per group) and male mice (B, n = 7/17 per group) during development. **(C, D)** Bar graphs showing body composition of adult Kiss1-Cre/Vgat*^flox/flox^*and control female (n = 4/5 per group) and male mice (n = 10/14 per group). Values are expressed as mean ± SEM. *P< 0.05, ***P<0.001 ****P<0.0001.

Assessment of the body weight gain during development showed a significant contribution of the genotype in both female and male Kiss1-Cre/Vgat*^flox/flox^* mice, so these animals exhibited an increased body weight gain compared to control mice throughout the post-weaning development period (female, *P=* 0.03; male, *P=* 0.0004; **Figures 4A, B**). However, no significant interaction between genotype and age was observed (female, *P=*0.9; male, *P=*0.9). In addition, 5-month-old Kiss1-Cre/Vgat*^flox/flox^* animals exhibited similar body weight, total fat, and lean mass compared to control mice (*P>*0.05; **Figures 4C, D**). Of note, gonad masses were similar between adult Kiss1-Cre/Vgat*^flox/flox^* and control animals (*P>*0.05; **Table 2**).

**Table 2:** The relative weight of the reproductive organs of adult mice.

Organs	Control	Kiss-Cre/Vgat *^flox/flox^*	*P* value
Uterus (g)	0.06 ± 0.01	0.06 ± 0.01	0.9
Ovarian (g)	0.005 ± 0.001	0.003 ± 0.001	0.1
Testicle (g)	0.08 ± 0.01	0.09 ± 0.01	0.4
Full seminal vesicle (g)	0.2 ± 0.0	0.2 ± 0.01	0.6

Values expressed as mean ± SEM. Females, n = 7/8 per group; males, n = 6/11 per group.

### Sexual maturation was not affected by *Vgat* ablation in kisspeptin cells

**Figure 5:**
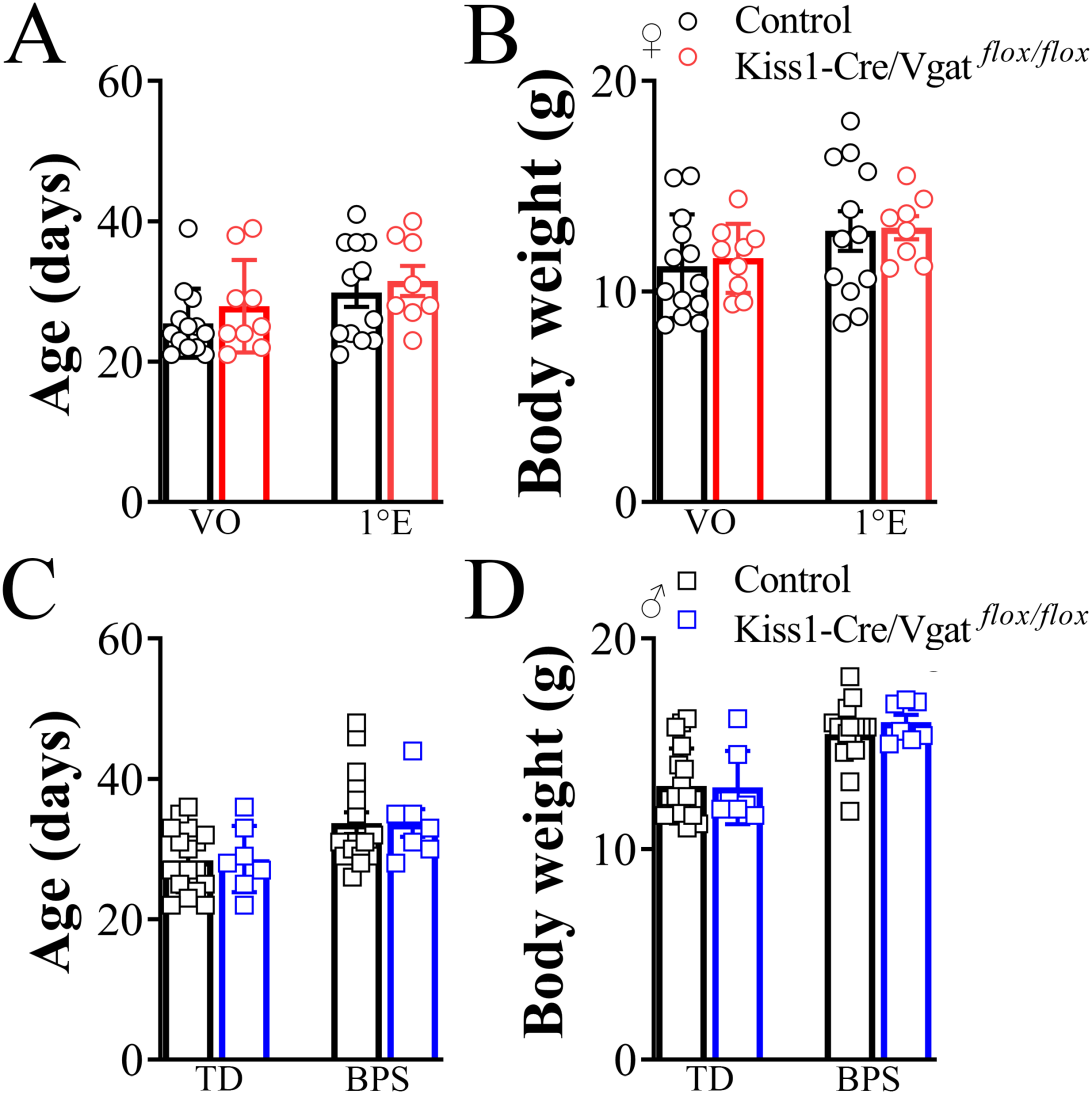
The vesicular GABA transporter ablation in kisspeptin cells does not impair sexual maturation of mice. **(A, B)** Bar graphs showing the average time required for female Kiss1-Cre/Vgat*^flox/flox^*and control mice to exhibit vaginal opening (VO), first estrus (1°E, A), and their body weight at specific stages of sexual maturation **(B)**. **(C, D)** Bar graphs showing the average time required for male Kiss1-Cre/Vgat*^flox/flox^*and control mice to exhibit testicular descent (TD), balanopreputial separation (BPS, C), and their body weight at specific stages of sexual maturation **(D)**. (females, n = 8/13 per group; males, n = 7/17 per group). Values are expressed as mean ± SEM. *P*>0.05.

Considering that *Vgat* conditional deletion from kisspeptin cells led to a downregulation of the *Gnrh1* and *Kiss1r* mRNA levels in the RPV of Kiss1-Cre/Vgat*^flox/flox^* mice, we next evaluated whether *Vgat* expression in kisspeptin cells contributes to puberty onset. The sexual maturation was assessed immediately after weaning. The average age of vaginal opening and first estrus of the Kiss1-Cre/Vgat*^flox/flox^* mice was similar compared to control animals (*P>*0.05; **Figure 5A**). No difference was found in the average body weight at the age of vaginal opening and first estrus by comparing the Kiss1-Cre/Vgat*^flox/flox^* to control mice (*P>*0.05; **Figure 5B**). Like the females, the Kiss1-Cre/Vgat*^flox/flox^* male mice exhibited similar puberty timing compared to control animals, as well as similar body weight at the specific ages in which balanopreputial separation and testicular descent were observed (*P>*0.05; **Figures 5C, D**).

### Fertility is preserved in Kiss1-Cre/Vgat^flox/flox^ mice

**Figure 6:**
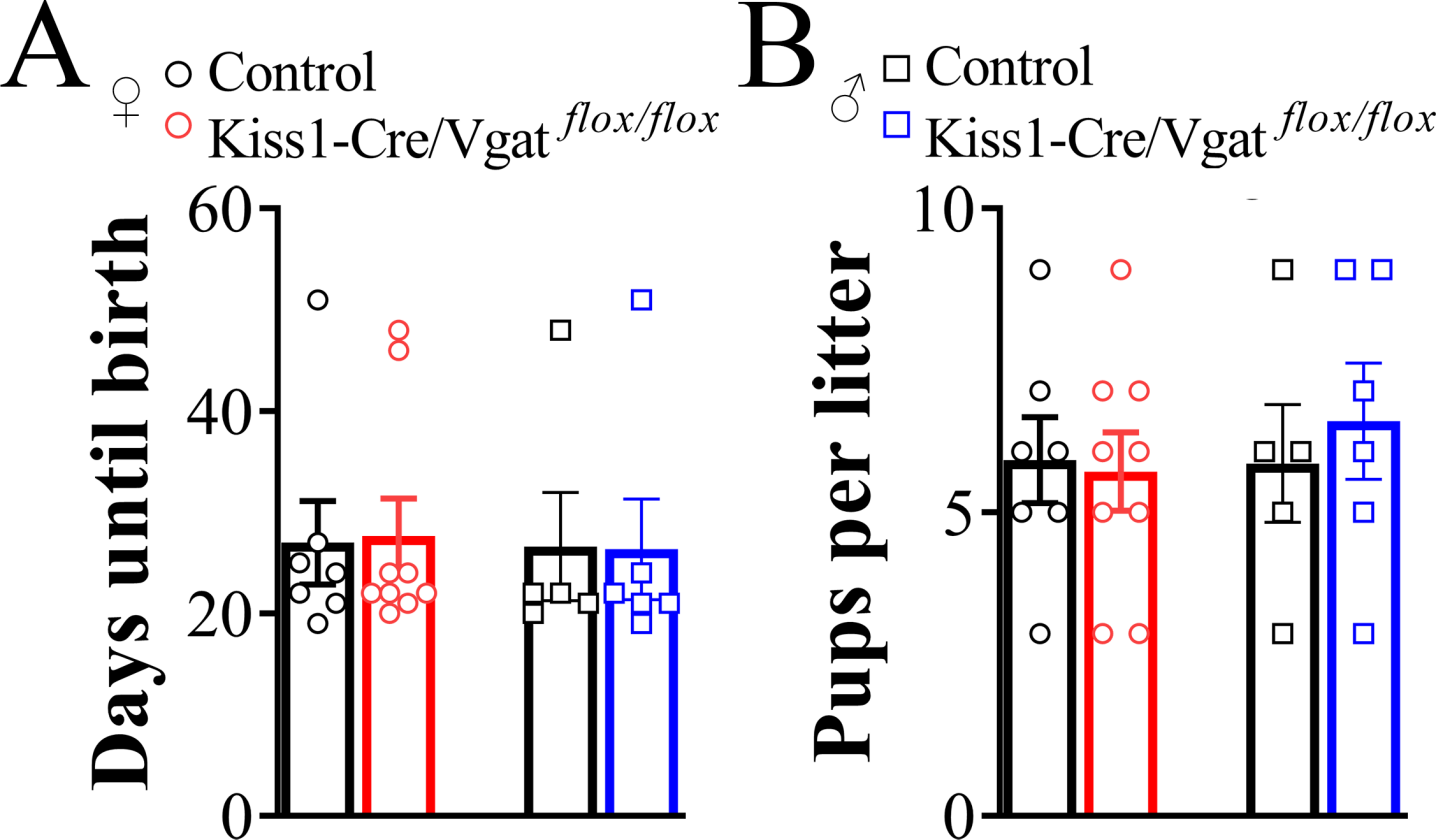
Vesicular GABA transporter expression in kisspeptin cells is not required for fertility. **(A, B)** Bar graphs showing the average latency to the first litter (days until birth, A) and the average number of pups per litter **(B)** in control (female, n = 7; male, n = 5 litters) and the Kiss1-Cre/Vgat*^flox/flox^*mice (female, n = 9; male, n = 6 litters). Values are expressed as mean ± SEM. *P*>0.05.

Fertility was evaluated by pairing Kiss-Cre/Vgat^flox/flox^ animals with sexually experienced mice. The proportion of Kiss1-Cre/Vgat*^flox/flox^* females that became pregnant and delivered pups (9 out of 10 mice) was similar to that observed in control females (7 out of 8 mice, *P*>0.9). Likewise, Kiss1-Cre/Vgat*^flox/flox^* males (6 out of 8 mice) successfully impregnated females at rates similar to those of control males (5 out of 6 mice, *P*>0.9). The latency to the first litter (**Figure 6A**) and the number of pups per litter (**Figure 6B**) did not differ between genotypes in either sex (*P>*0.05). Together, these findings indicate that fertility is preserved in both female and male Kiss1-Cre/Vgat*^flox/flox^* mice.

### *Vgat* expression in kisspeptin cells may contribute to the preovulatory LH surge

**Figure 7:**
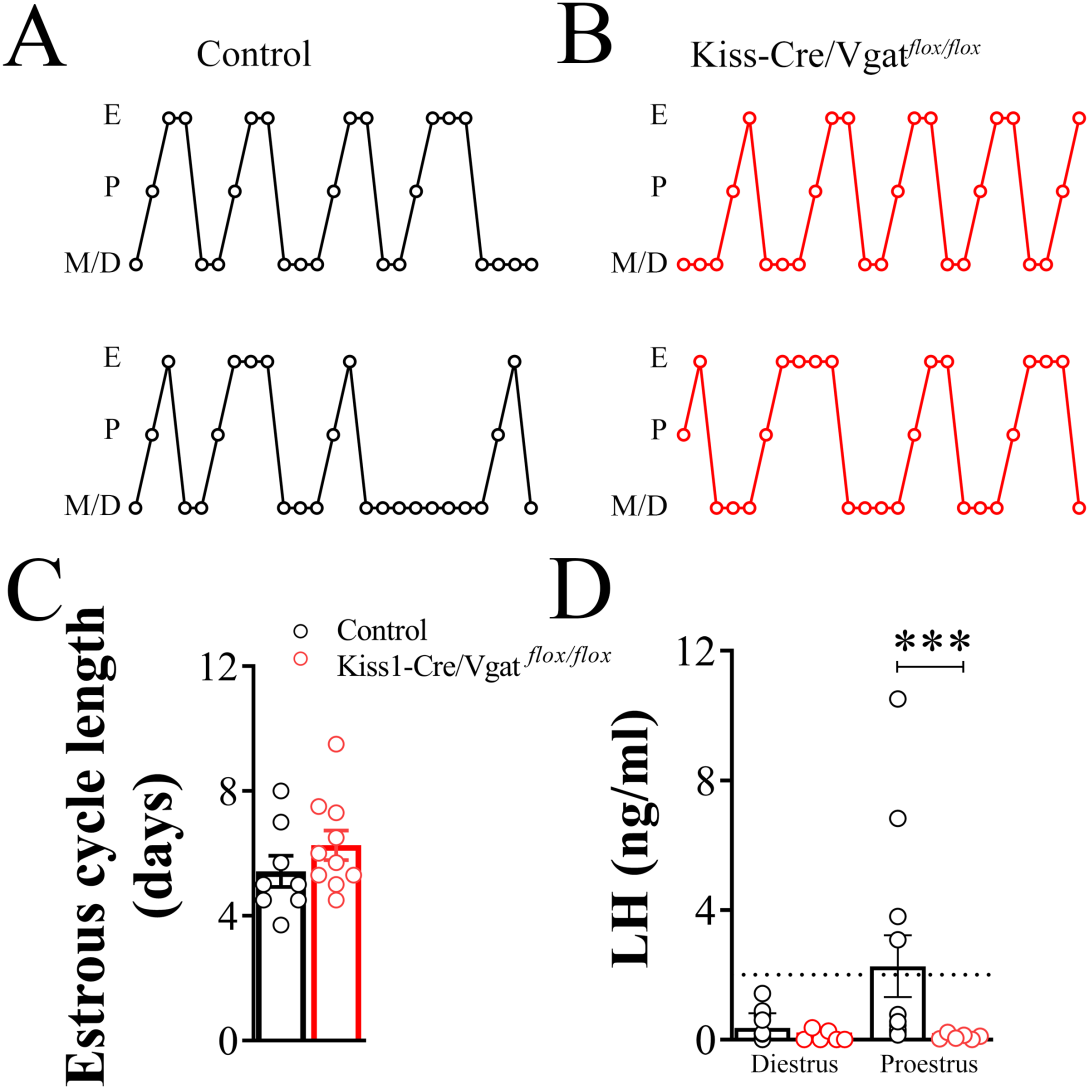
Vesicular GABA transporter expression in kisspeptin cells contributes to the preovulatory gonadotropin surge. **(A, B)** Representative estrous cycle profiles of control and Kiss1-Cre/Vgat*^flox/flox^* mice. **(C)** Bar graphs showing the average estrous cycle length of the control (n = 8) and the Kiss1-Cre/Vgat*^flox/flox^* mice (n = 10). A complete estrous cycle was defined as the occurrence of all estrous stages in sequence, starting on the first day of diestrus. **(D)** Average luteinizing hormone (LH) serum levels in the control and the Kiss1-Cre/Vgat*^flox/flox^* female mice at the diestrus and proestrus (n =6/12 per group). Values are expressed as mean ± SEM. ***P=0.0003.

Estrous cyclicity was assessed in adult female Kiss1-Cre/Vgat*^flox/flox^* and control mice. Cycle length did not differ between groups (**Figures 7A–C**). Serum LH concentrations during diestrus were comparable between genotypes (n=6/12 per group; *P* = 0.1; **Figure 7D**). In contrast, during the afternoon of proestrus, Kiss1-Cre/Vgat*^flox/flox^* mice exhibited reduced serum LH levels compared with control mice (n =6/12 per group; *P* = 0.0003; **Figure 7D**). This reduction was attributable to the absence of elevated LH concentrations (>2 ng/mL) in Kiss1-Cre/Vgat^flox/flox^ mice (0 out of 6 animals), whereas 4 of 12 control mice exceeded this threshold (**Figure 7D**). However, no statistically significant difference in the number of mice exhibiting an LH surge was observed between groups (P = 0.2).

## Discussion

Collectively, our data indicate that *Vgat* expression in kisspeptin cells may contribute to sex steroid-mediated regulation of the preovulatory surge. This conclusion is supported by the observed downregulation of *Gnrh1* and *Kiss1r*, together with a reduced LH surge during the afternoon of proestrus. Nevertheless, the overall effects were modest, suggesting a subtle contribution to sex steroid-dependent control of the preovulatory surge. Consistent with this interpretation, *Vgat* expression in kisspeptin cells was not required for sexual maturation or fertility in either female or male mice.

Approximately 50-60% of AVPV/PeN^Kisspeptin^ neurons exhibit a GABAergic phenotype, as demonstrated in this study and others (34). Although the proportion of AVPV/PeN^Kisspeptin^ *Vgat-*expressing cells appears to remain stable during the diestrus and proestrus phases, it is important to note that the Cre-lox system does not allow for the detection of dynamic changes caused by the fluctuations of gonadal hormone levels across the estrous cycle; therefore, we cannot exclude the possibility that variations in *Vgat* expression occur in the AVPV/PeN or even in the ARH neurons. While the majority of ARH^Kisspeptin^ neurons are glutamatergic, a subset has been reported to co-express glutamic acid decarboxylase GAD67 and *Vgat* (34, 35, 46). However, unlike the AVPV/PeN population, the neurochemical profile of ARH^Kisspeptin^ cells could not be assessed in the present study.

By generating the Kiss1-Cre/Vgat*^flox/flox^* mouse model, we were able to investigate the functional significance of *Vgat* expression in kisspeptin-producing cells for the regulation of reproductive functions. The efficiency of *Vgat* deletion was assessed using two complementary approaches. A significant reduction in *Vgat* mRNA expression was observed in AVPV/PeN^Kisspeptin^ neurons from Kiss1-Cre/Vgat*^flox/flox^* mice, and *Vgat* levels were significantly reduced in microdissected RPV tissue, indicating efficient Cre-mediated recombination. In contrast, no significant reduction in *Vgat* expression was observed in MBH punches. The persistence of residual *Vgat* mRNA expression was expected, given the presence of non-kisspeptin GABAergic neurons within these regions (42). Given the conflicting reports in the literature regarding the proportion of ARH^Kisspeptin^ cells that co-express *Vgat* (35, 46), and because we did not directly assess *Kiss1/Vgat* co-expression in ARH cells in the present study, it remains unclear to what extent the neurochemical phenotype of ARH^Kisspeptin^ neurons was modified in the Kiss1-Cre/Vgat*^flox/flox^* mouse model. This represents a limitation of the current study. While previous studies have highlighted a fundamental role for ARH GABAergic cells in the control of the HPG axis, as chronicactivation of these neurons disrupts estrous cyclicity, reduces the number of corpora lutea, and increases testosterone levels in female mice (55), the reproductive phenotype of Kiss1-Cre/Vgat*^flox/flox^*, together with the preservation of the estrous cyclicity and fertility, argues against a major contribution of GABAergic-ARH^Kisspeptin^ neurons to the phenotype observed in our study.

In addition, many studies have explored the role of ARH^Kisspeptin^ in modulating metabolism (20–24). In our study, both female and male knockout mice exhibited a modest but statistically significant increase in body weight gain across development. However, no significant differences were detected at specific ages, nor in adult fat and lean mass composition. Although changes in energy homeostasis modulate GABAergic transmission to ARH^Kisspeptin^ neurons (22), *Vgat* expression in kisspeptin cells appears to have a modest influence on body weight gain, at least in mice maintained on a standard diet.

The fertile phenotype of Kiss1-Cre/Vgat*^flox/^*^flox^ mice was not surprising, considering that previous studies have demonstrated that HPG axis maturation and fertility can be maintained despite reduced *Kiss1* or *Gnrh1* mRNA expression (56–59). Mice still undergo expected puberty timing, exhibit regular sex-steroid modulation of the ovulatory cycle, and are fertile even without 95% of the kisspeptin neurons (59). A similar phenotype was seen in different mouse models in which *Kiss1* mRNA expression was downregulated, or in which the GABA_A_ receptor was conditionally deleted from GnRH neurons (52, 57, 60–63). Likewise, an expressive reduction in the number of GnRH neurons or in *Gnrh1* expression had no effect on sexual maturation in mice (58). In our study, because *Vgat* ablation occurred during development, we cannot rule out the possibility that compensatory mechanisms maintained reproductive function.

Our model further aligns with previous studies investigating the role of GABAergic signaling in mediating the effects of sex steroids on the ovulatory cycle (42, 44). CRISPR-mediated *Esr1* knockdown in GABAergic neurons in the preoptic area results in variable reproductive phenotypes, with most mice exhibiting regular estrous cycles and LH secretion (44). Mice lacking kisspeptin immunoreactivity in the AVPV/PeN fail to demonstrate an LH surge but retain pulsatile LH secretion. In contrast, a broader lack of *Esr1* signaling in GABA neurons, including AVPV/PeN^Kisspeptin^ cells, leads to infertility, abnormal estrous cycles, and failure of the estrogen-positive feedback mechanism responsible for the preovulatory GnRH surge, while puberty onset occurs normally (42). In addition, we have previously demonstrated that AVPV/PeN^Kisspeptin^ neurons, alone, in the absence of ARH^Kisspeptin^ cells due to a monosodium glutamate-induced lesion, are sufficient to induce puberty (49). Accordingly, although approximately 50-60% of AVPV/PeN^Kisspeptin^ cells are GABAergic, *Vgat* ablation in kisspeptin cells did not impair puberty timing in the Kiss1-Cre/Vgat*^flox/flox^* mice, despite reduced *Kiss1r* and *Gnrh1* mRNA levels. The absence of major effects on estrous cyclicity in Kiss1-Cre/Vgat*^flox/^*^flox^ mice is further consistent with previous evidence indicating that GABA release from AVPV/PeN^kisspeptin^ neurons may be functionally redundant, while kisspeptin signaling remains the predominant driver of GnRH neuron output (47).

It is also well established that not all female mice within an experimental cohort exhibit an LH surge within a narrow proestrus time window, as reported by independent groups, including ours (22, 53, 64). Because LH measurements were obtained from single-time-point samples collected during the afternoon of proestrus, a shift in the timing of the LH cannot be excluded and may have contributed to the observed results.

## Conclusions

Our data demonstrate that *Vgat* expression in kisspeptin cells modestly contributes to the preovulatory gonadotropin surge, as evidenced by the impact on *Gnrh1* and *Kiss1r* mRNA expression and the impaired LH surge in the afternoon of proestrus. However, conditional *Vgat* ablation in kisspeptin cells does not affect puberty or fertility in both female and male mice. These findings collectively contribute to understanding the intricate mechanisms involving kisspeptin and GABAergic signaling in the control of reproduction.

## Data Availability

The data supporting this study's findings are available from the corresponding author upon reasonable request.
